# Dram1 regulates DNA damage-induced alternative autophagy

**DOI:** 10.15698/cst2018.03.127

**Published:** 2018-03-07

**Authors:** Meruna Nagata, Satoko Arakawa, Hirofumi Yamaguchi, Satoru Torii, Hazuki Endo, Masatsune Tsujioka, Shinya Honda, Yuya Nishida, Akimitsu Konishi, Shigeomi Shimizu

**Affiliations:** 1 Department of Pathological Cell Biology, Medical Research Institute, Tokyo Medical and Dental University, 1-5-45 Yushima, Bunkyo-ku, Tokyo 113-8510, Japan.; 2 Present address: Department of Biochemistry, Gunma University Graduate School of Medicine, 3-39-22 Showa, Maebashi, Gunma 371-8511 Japan.

**Keywords:** alternative autophagy, DNA damage response, genotoxic stress, p53, Dram1

## Abstract

Autophagy is an evolutionarily conserved process that degrades subcellular constituents. Mammalian cells undergo two types of autophagy; Atg5-dependent conventional autophagy and Atg5-independent alternative autophagy, and the molecules required for the latter type of autophagy are largely unknown. In this study, we analyzed the molecular mechanisms of genotoxic stress-induced alternative autophagy, and identified the essential role of p53 and damage-regulated autophagy modulator (Dram1). Dram1 was sufficient to induce alternative autophagy. In the mechanism of alternative autophagy, Dram1 functions in the closure of isolation membranes downstream of p53. These findings indicate that Dram1 plays a pivotal role in genotoxic stress-induced alternative autophagy.

## INTRODUCTION

Macroautophagy (hereafter described as autophagy) is a cellular mechanism by which cells digest their own cellular components, including proteins, lipids, and even entire organelles. The molecular mechanisms of autophagy have been extensively studied regarding starvation-induced autophagy, in which autophagy-related (Atg) proteins induce the formation of autophagosomes and autolysosomes 1,2. In the initial step of starvation-induced autophagy, Unc-51-like kinase 1 (Ulk1), a homologue of yeast Atg1, translocates to preautophagosomal membranes and forms a multi-protein complex together with Fip200, Atg13, and Atg101, in a manner dependent on Ulk1 dephosphorylation 3,4,5. The Ulk1 complex activates puncta formation of double-FYVE-containing protein 1 and generates the omegasome, from which the isolation membrane grows out. Subsequent expansion and closure of isolation membranes are mediated by two ubiquitin-like conjugation pathways, namely, the Atg5-Atg12 pathway and the microtubule-associated protein 1 light chain 3 (LC3) pathway. By the fusion of autophagosomes and lysosomes, cargos are degraded within autolysosomes 1,2. Among the various components involved in autophagy, several molecules, particularly Atg5 and Atg7, have long been believed to be indispensable.

However, in addition to Atg5/Atg7-dependent autophagy, we discovered an alternative type of autophagy that occurs even in cells lacking Atg5 and Atg7 6. The morphology and biochemical functions of this alternative autophagy are similar to Atg5/Atg7-dependent conventional autophagy; i.e., cellular components and organelles are enclosed by autophagosomes (double-membrane vesicles) and are subsequently digested within autolysosomes. However, these two autophagy pathways are used differently in stimulus-dependent and context-dependent manners; i.e., starvation induces only the conventional type whereas genotoxic stress induces both types 6. Furthermore, in the terminal differentiation of erythrocytes, alternative and conventional autophagy eliminate mitochondria and ribosomes, respectively 7.

Most of the molecules required for the conventional autophagy have been identified and characterized. In contrast, only a few molecules associated with alternative autophagy have been identified. One such molecule is Ulk1, which drives the initial steps of both types of autophagy 6. In contrast, Rab9 functions in the elongation and closure of the isolation membrane only in alternative autophagy 6. Many more molecules are thought to be required for alternative autophagy, and hence we here searched for such molecules. To identify molecules required for alternative autophagy, we subjected Atg5-knockout cells to genotoxic stress, which is a strong inducer of alternative autophagy, and analyzed the role of p53, because p53 is a master regulator of genotoxic stress. We found that genotoxic stress-induced alternative autophagy occurs in a p53-dependent manner. Furthermore, we demonstrate that damage-regulated autophagy modulator (Dram1), which is a molecule induced by p53 8,9, is necessary and sufficient to induce alternative autophagy. We also suggest that Dram1 functions in the closure of the isolation membranes.

## RESULTS 

**Figure 1:**
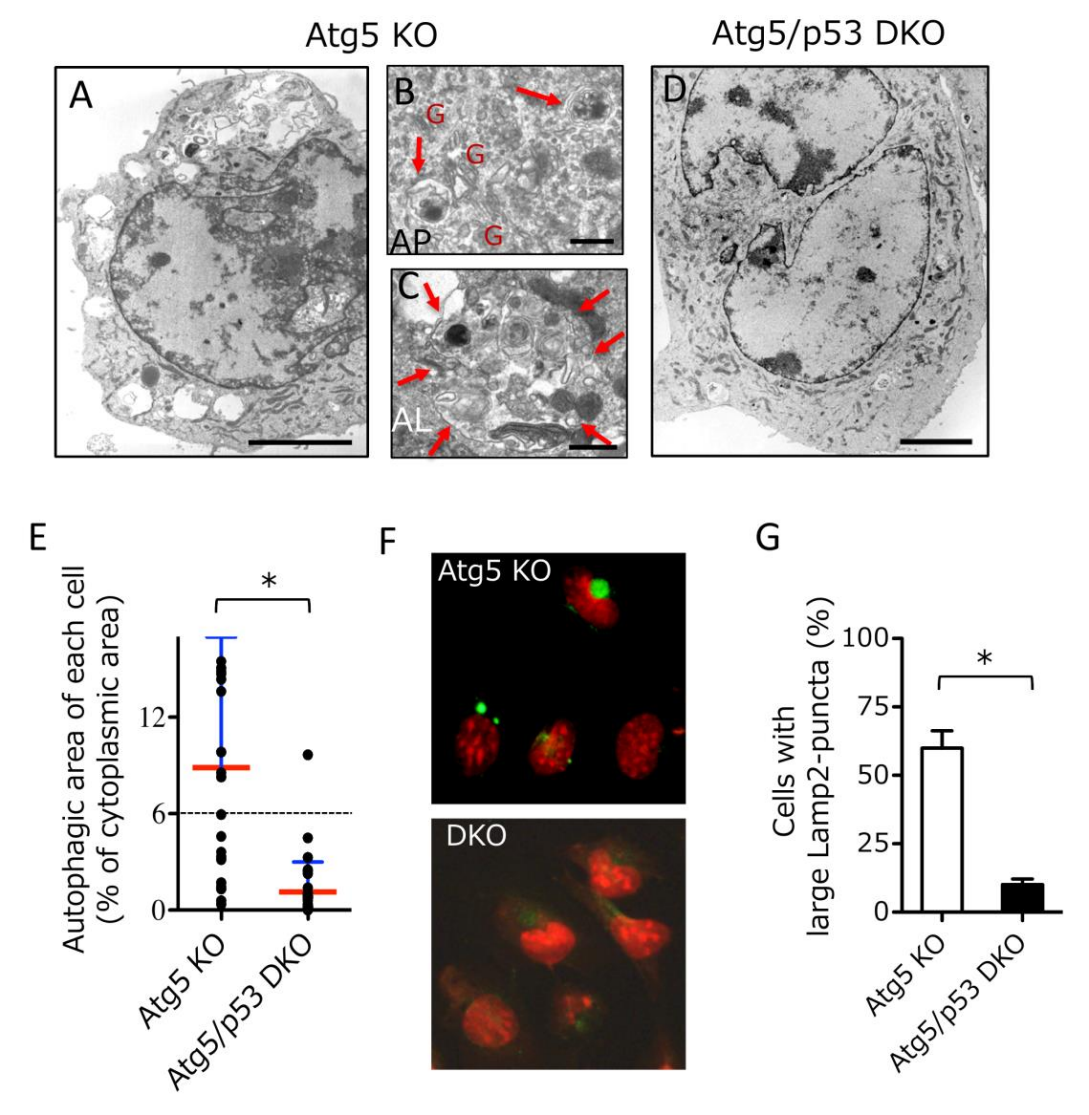
FIGURE 1: Induction of autophagy by etoposide in Atg5 KO MEFs, but not Atg5/p53 DKO MEFs. **(A-D) **Electron micrographs of Atg5 KO MEFs **(A-C)** and Atg5/p53 DKO MEFs **(D) **treated with etoposide (10 μM) for 18 h. In** (A, D)**, bar = 5 μm. In** (B)**, arrows indicate autophagosomes. "G" indicates Golgi membranes. Bar = 0.5 μm. In** (C)**, the arrows are surrounding an autolysosome. Bar = 0.5 μm. **(E)** The percentage autophagic area of MEFs treated with etoposide. The indicated MEFs were treated with etoposide for 18 h and the autophagic area of each cell was calculated (n > 25 cells each). Red and blue lines indicate the mean and SD, respectively. The dotted line indicates the autophagic threshhold. *p < 0.05. **(F)** The indicated MEFs were treated with etoposide (10 μM) for 12 h. The cells were then examined for Lamp2 immunofluorescence (green) and nuclei were counterstained with propidium iodide (red). **(G) **The percentage of cells with large Lamp2 puncta is shown as the mean + SD (n = 4). *p < 0.05.

To clarify the molecular mechanisms of genotoxic stress-induced alternative autophagy, we investigated the requirement of p53, because p53 is a molecule involved in various genotoxic stress-induced responses 10,11,12, including apoptosis and conventional autophagy. To this end, we generated Atg5/p53 double-knockout (DKO) mouse embryos by crossbreeding p53 heterozygous knockout (KO) mice with Atg5 heterozygous KO mice and isolated their mouse embryonic fibroblasts (MEFs). We added etoposide, a DNA-damaging reagent that inhibits topoisomerase II, into the culture medium of Atg5/p53 DKO MEFs and littermate Atg5 KO MEFs, and analyzed alternative autophagy using electron microscopy (EM). As described previously 6, numerous autophagic vacuoles were observed in Atg5 KO MEFs (**Fig. 1A**). Representative autophagosomes (double-membrane vesicles) (**Fig. 1B**) and autolysosomes degrading cellular constituents (**Fig. 1C**) were observed. However, we did not observe any such structures in Atg5/p53 DKO MEFs (**Fig. 1D**). Quantitative analysis of autophagic area per cell confirmed the induction of alternative autophagy by etoposide exposure in Atg5 KO MEFs, but not Atg5/p53 DKO MEFs (**Fig. 1E**). Note that autophagic cells were defined as cells in which autophagic vacuoles exceed 6% of the cytoplasmic area, which was the upper limit in untreated healthy cells. Alternative autophagy can be assessed more easily by the immunostaining of the lysosomal protein Lamp2 (or Lamp1), because lysosomal fluorescence is usually seen as small dots that become large puncta by the induction of autophagy (owing to the fusion of lysosomes with autophagic vacuoles). The correspondence of large Lamp2 (or Lamp1) puncta to autolysosomes was previously shown by correlative light electron microscopy (CLEM) analysis 6, and was also confirmed in the present study (see **Fig. 3D**). Using the Lamp2 immunostaining assay, we observed large Lamp2 puncta (autolysosomes) in Atg5 KO MEFs, but not Atg5/p53 DKO MEFs (**Fig. 1F, G**), consistent with the EM observations (**Fig. 1E**). These results indicated that p53 is necessary for the induction of etoposide-induced alternative autophagy.

**Figure 2:**
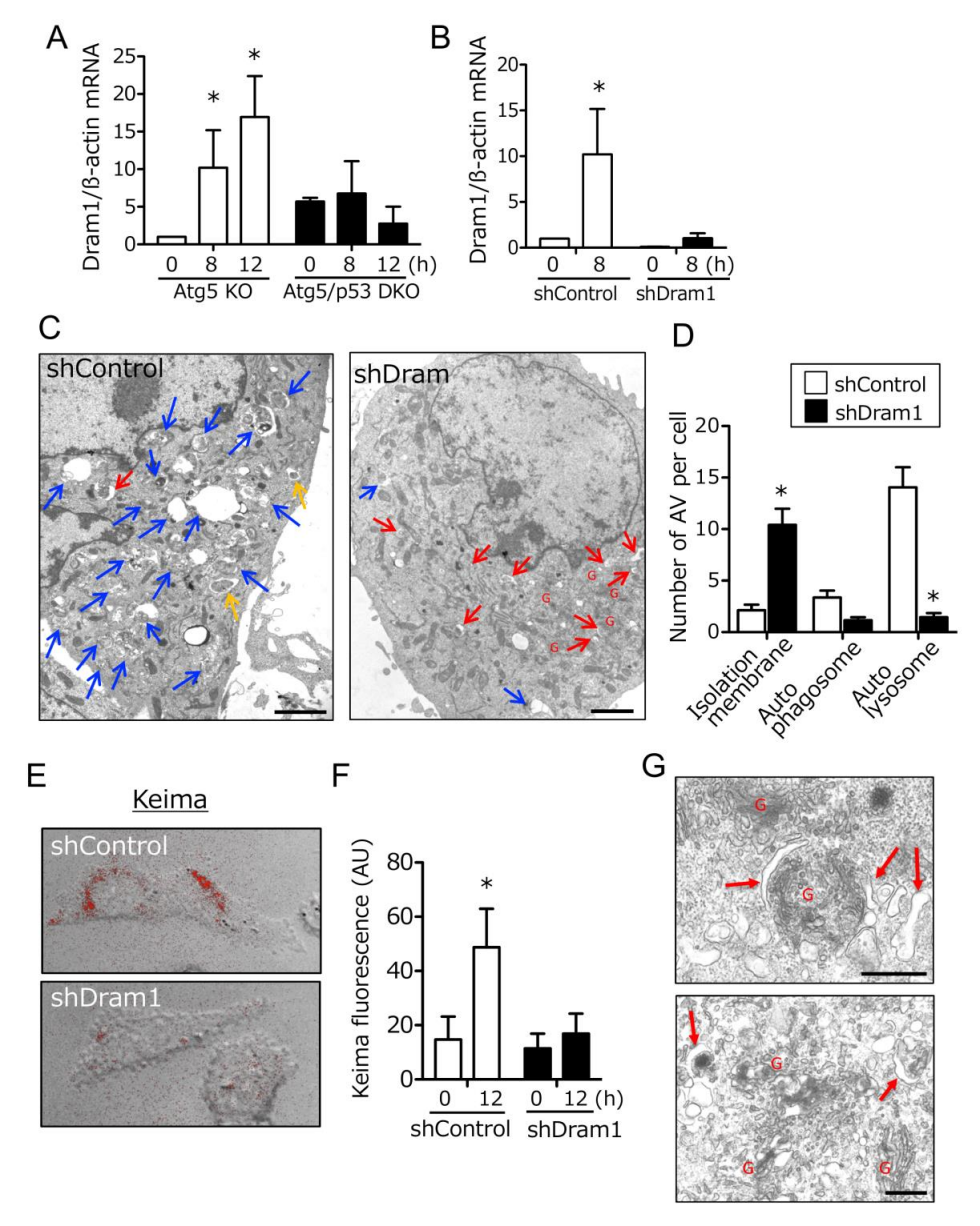
FIGURE 2: Inhibition of alternative autophagy in etoposide-treated Atg5 KO MEFs by an shRNA for Dram1. **(A, B)** Dram1 expression was assessed by qPCR using mRNA.** (A) **Atg5 KO MEFs and Atg5/p53 DKO MEFs were incubated with 10 μM etoposide for the indicated times. *p < 0.05 vs the value of "Atg5 KO 0 h".** (B) **shDram1-transfected and control Atg5 KO MEFs were incubated with 10 μM etoposide for the indicated times. *p < 0.05 vs the value of "shControl 0 h". **(C, D) **Electron micrograph of shDram1-transfected Atg5 KO MEFs and control Atg5 KO MEFs treated with etoposide (10 μM) for 18 h. Blue, yellow, and red arrows indicate autolysosomes, autophagosomes, and isolation membranes, respectively. "G" indicates Golgi membranes. In **(C)**, bar = 2 μm. **(D) **The number of each type of autophagic structure appearing in Atg5 KO MEFs treated with etoposide. shDram1-transfected and control Atg5 KO MEFs were incubated with 10 μM etoposide for 18 h, and the number of autophagic vacuoles per cell was counted on the EM photographs. White and black columns represent the number of autophagic structures in control and shDram1-transfected Atg5 KO MEFs, respectively. Data are the mean + SD obtained from 10 cells. *p < 0.05 vs the value of "shControl".** (E, F)** Keima analysis indicated the requirement of Dram1 in etoposide-induced alternative autophagy. The indicated MEFs were treated with etoposide (10 μM) for 12 h. Alternative autophagy was then analyzed using Keima. **(E) **Keima signals (red) were merged with images obtained from phase-contrast microscopy at 12 h. **(F)** The extent of Keima fluorescence is shown as the mean + SD (n = 4). *p < 0.05 vs the value of "shControl 0 h". **(G) **Electron micrograph of shDram1-transfected Atg5 KO MEFs treated with etoposide (10 μM) for 18 h. Red arrows indicate isolation membranes. "G" indicates Golgi membranes. Bar = 0.5 μm.

Because p53 is a transcription factor, molecules that are transcriptionally upregulated by p53 are expected to be crucial for alternative autophagy. Thus, we compared gene expression profiles of Atg5 KO MEFs and Atg5/p53 DKO MEFs after etoposide treatment, and identified Dram1 as one of the most upregulated molecules in Atg5 KO MEFs, but not Atg5/p53 DKO MEFs. We confirmed this upregulation by real-time PCR (**Fig. 2A**), given that no anti-mouse Dram1 antibody for Western blotting was available. Dram1 has already been reported as a molecule involved in conventional autophagy 8,9. To address whether Dram1 is also required for etoposide-induced alternative autophagy, we created stable Dram1-silenced MEFs and control MEFs from Atg5 KO MEFs by the transfection of short hairpin RNA (shRNA). Dram1 was not increased after etoposide treatment in Dram1-silenced MEFs (**Fig. 2B**). When we assessed etoposide-induced alternative autophagy using EM, many autolysosomes were observed in control Atg5 KO MEFs (**Fig. 2C**; blue arrows, 2D), whereas such structures were not observed in Dram1-silenced Atg5 KO MEFs (**Fig. 2C, D**). Consistent results were also observed when Atg5 KO MEFs were transiently transfected with different siRNA for Dram1 (Suppl. Fig. 1A, B). The crucial role of Dram1 in alternative autophagy was also shown by another autophagy assay using Keima. Keima is a fluorescent protein that enables the detection of autolysosomes by its emission of different-colored signals at acidic and neutral pHs 13. By the expression of Keima followed by etoposide treatment, we observed autolysosomal signals around the nucleus in control Atg5 KO MEFs, but not in stable Dram1-silenced Atg5 KO MEFs (**Fig. 2E, F**), consistent with the EM observations (**Fig. 2D**). Detailed EM analyses of etoposide-treated Atg5 KO MEFs expressing shDram1 showed a large number of curved and swollen isolation membranes which were at a step before their enclosure of subcellular constituents (**Fig. 2G**). These membranes were localized close to the Golgi membrane (**Fig. 2G**). This morphology suggested that etoposide-induced alternative autophagy originates from Golgi membranes, and that loss of Dram1 blocks autophagosome generation by inhibiting the elongation and closure of isolation membranes. Note that camptothecin, another DNA-damaging reagent that induces genotoxic stress via a mechanism different from etoposide, also induced Dram1-dependent alternative autophagy (Suppl. Fig. 2). In contrast, staurosporine (STS), a pan-kinase inhibitor, induced alternative autophagy, but it was not affected by Dram1 silencing (Suppl. Fig. 3). Therefore, Dram1 is only crucial for genotoxic stress-induced alternative autophagy.

**Figure 3:**
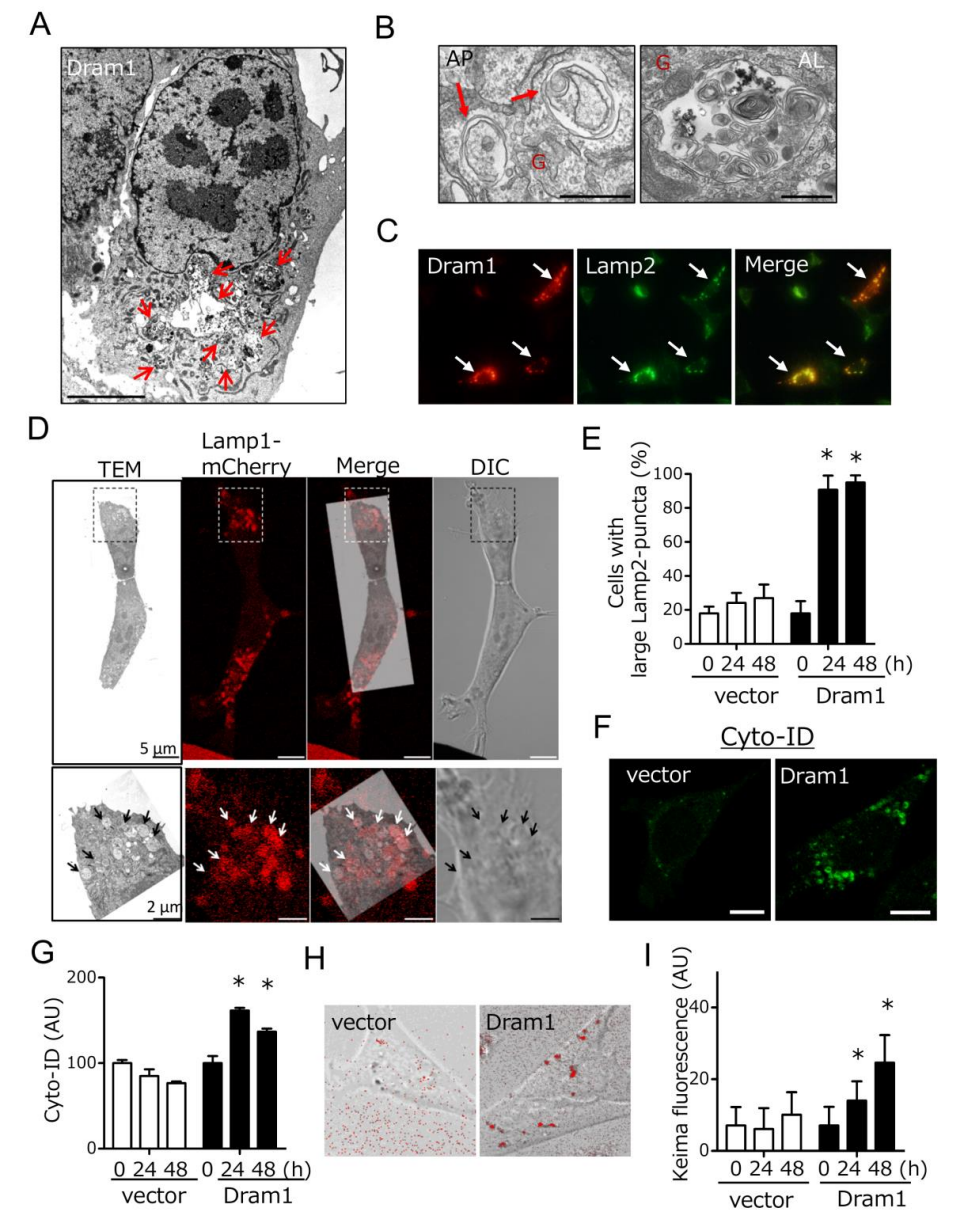
FIGURE 3: Induction of alternative autophagy in Atg5 KO MEFs by Dram1 expression. **(A, B)** Electron micrographs of Atg5 KO MEFs 12 h after the transfection of *dram1-flag*. In **(A)**, arrows indicate autolysosomes. Bar = 5 μm. In **(B)**, a representative autophagosome (AP, left) and autolysosome (AL, right) are shown. Arrows indicate autophagosomes. "G" indicates Golgi membranes. Bar = 0.5 μm. **(C-E)** Induction of alternative autophagy by Dram1 was assessed by Lamp2 immunostaining. In **(C)**, Atg5 KO MEFs were transfected with *dram1-flag* for 24 h. The cells were then analyzed for immunofluorescence of Lamp2 and Flag. Arrows indicate Atg5 KO MEFs in which Dram1 was efficiently transfected. **(D)** Identification of autolysosomes as Lamp1-positive structures by CLEM. Lamp1-mCherry-expressing Atg5 KO MEFs were transfected with *dram1-flag*, and were incubated for 24 h. Then CLEM analysis was performed. The magnified images of the dashed squares are shown in the bottom panels. Lamp1-positive structures (red signals; arrows) were identical to large autolysosomes. **(E)** Atg5 KO MEFs were transfected with *dram1-flag* or control vector for the indicated times. The cells were then analyzed for immunofluorescence of Lamp2 and Flag. The percentage of cells with punctate Lamp2 immunofluorescence is shown as the mean + SD (n = 4). *p < 0.05 vs the value of "vector 0 h". **(F-I)** Induction of alternative autophagy in etoposide-treated Atg5 KO MEFs by Dram1. Atg5 KO MEFs were transfected with *dram1-flag* or control vector for the indicated times. Alternative autophagy was then analyzed using Cyto-ID **(F, G)** and Keima **(H, I)**. In **(F)** and** (H)**, representative images at 24 h are shown. The extent of Cyto-ID fluorescence** (G)** and Keima fluorescence **(I)** is shown as the mean + SD (n = 4). *p < 0.05 vs the value of "vector 0 h".

We next analyzed whether Dram1 is not only necessary but also sufficient to induced alternative autophagy. To this end, we expressed Flag-tagged Dram1 in Atg5 KO MEFs, and analyzed alternative autophagy using EM. We observed many autophagic vacuoles (**Fig. 3A**), including representative autophagosomes (double-membrane compartments enclosing cargo) and autolysosomes (single-membrane compartments digesting cargo) (**Fig. 3B**) in Dram1-overexpressing Atg5 KO MEFs. Autophagosomes were localized close to the Golgi membrane (**Fig. 3B**). Analyses using Lamp2 immunofluorescence demonstrated that only cells efficiently expressing Dram1 showed large Lamp2 puncta formation (**Fig. 3C**; arrows), and these large puncta were actually merged with autolysosomal structures as assessed by the CLEM analysis (**Fig. 3D**). Furthermore, the number of large Lamp2 puncta-containing cells increased in a time-dependent manner in Dram1-expressing Atg5 KO MEFs, but not control MEFs (**Fig. 3E**), indicating that the induction of Dram1 is sufficient to induce alternative autophagy. We also analyzed whether Dram1 was sufficient to induce alternative autophagy using Cyto-ID, which is a novel dye that selectively labels accumulated autophagic vacuoles. CLEM analysis confirmed that Cyto-ID puncta are identical to autophagic vacuoles (Suppl. Fig. 4). As shown in **Fig. 3F** and **3G**, Cyto-ID puncta were observed upon the overexpression of Dram1 in Atg5 KO MEFs, and their intensity increased in a time-dependent manner. Keima analysis showed consistent results (**Fig. 3H, I**), indicating that the simple induction of Dram1 is sufficient to induce alternative autophagy.

**Figure 4:**
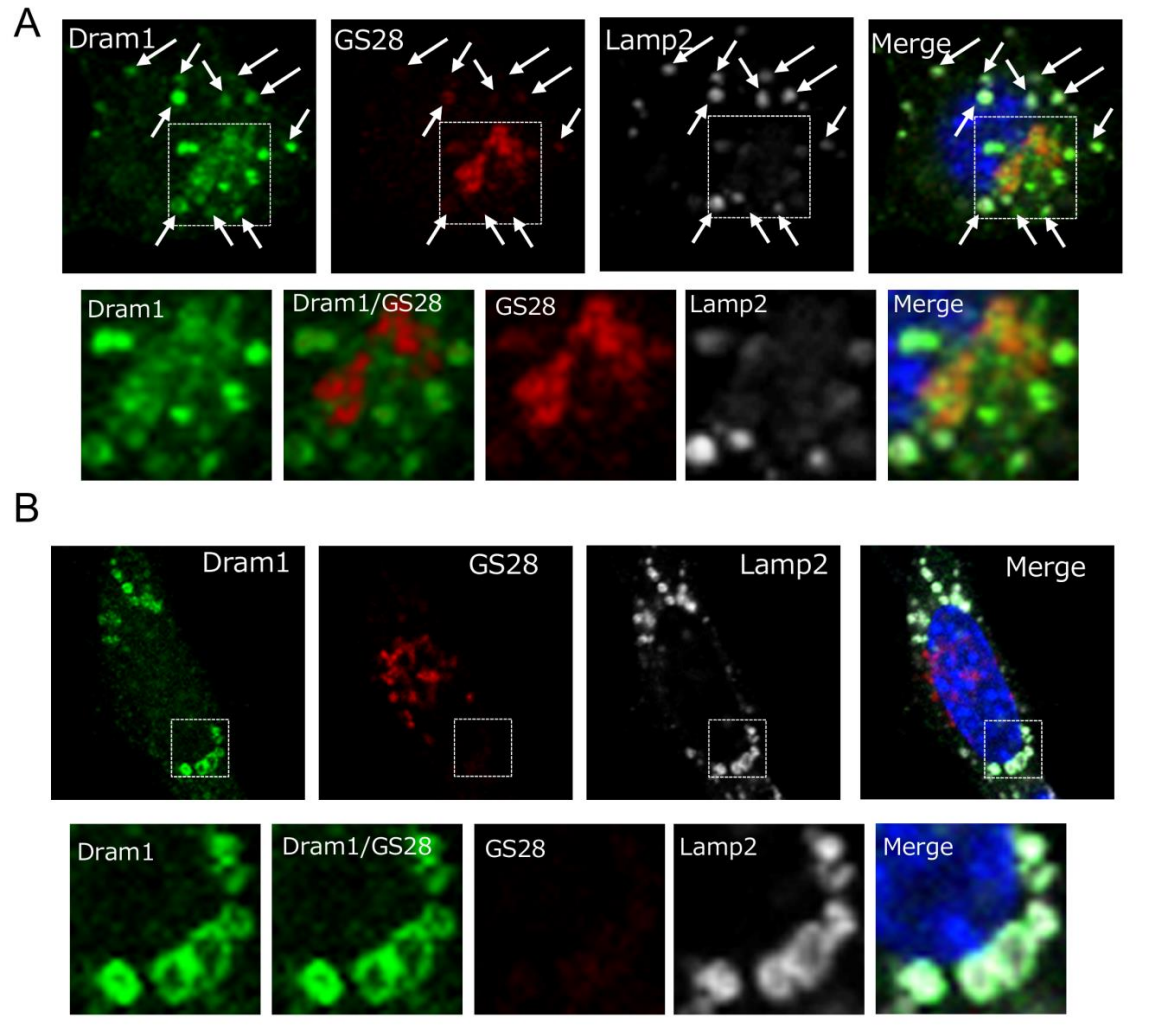
FIGURE 4: Intracellular localization of Dram1-Flag in Atg5 KO MEFs. Atg5 KO MEFs were transfected with *dram1-flag* for 24 h **(A)** and 48 h **(B)**. The cells were then examined for immunofluorescence of GS28 (a pan-Golgi marker), Lamp2 (a lysosome marker), and Flag (Dram1). Nuclei were counterstained with DAPI. In **(A)**, some Dram1 particles were merged with GS28 signals (dashed squares). Magnified images of the area within the dashed squares are shown in the bottom panels. Arrows indicate areas of Dram1 and Lamp2 colocalization. In **(B)**, none of the Dram1 particles were merged with GS28 signals (dashed squares). Magnified images of the area within the dashed squares are shown in the bottom panels.

Dram1 is a hydrophobic protein with 6 transmembrane domains, and was reported to localize on the Golgi membrane, early and late endosomes, and lysosomes 9. Consistently, some Flag-Dram1 particles merged with the pan-Golgi marker GS28 (**Fig. 4A**, dashed squares), whereas others merged with Lamp2 (**Fig. 4A**, arrows) at 12-24 hours after Dram1 overexpression. This Golgi localization is reasonable because Dram1 functions in the closure of isolation membranes, which originate from the Golgi membrane (**Fig. 2G**), and Dram1 generates autophagosomes next to the Golgi membrane (**Fig. 3B**). At 48 hours after transfection, most Dram1 signals were separate from GS28 signals, and were completely merged with ring-like structures of Lamp2, namely, autolysosomes (**Fig. 3C, 4B**).

**Figure 5:**
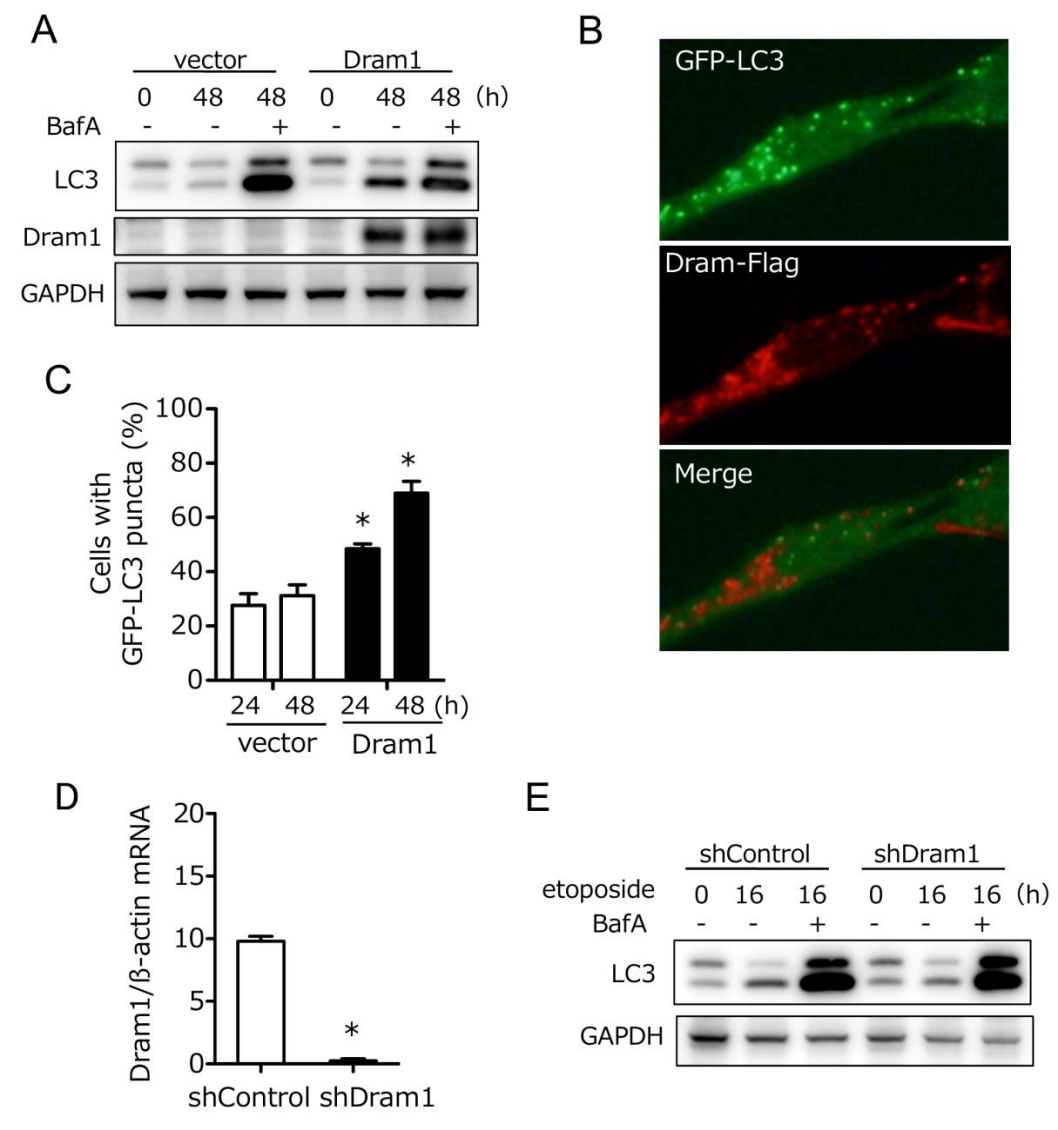
FIGURE 5: Dram1 is sufficient to induce conventional autophagy, but is not involved in etoposide-induced conventional autophagy. **(A-C)** Dram1 is sufficient to induce conventional autophagy. **(A) **WT MEFs were transfected with *dram1-flag* or a control vector for the indicated times in the presence or absence of bafilomycin A1 (BafA; 10 nM), and the expression of each protein was analyzed by western blotting. GAPDH was included as a loading control. **(B, C)** GFP-LC3-expressing WT MEFs were transfected with *dram1-flag* or control vector for the indicated times, and then immunostained with an anti-Flag antibody (red). GFP-LC3 signals are shown in green. Representative images at 24 h are shown in** (B)**. **(C) **The proportion of cells with LC3 puncta was calculated (n > 100 cells in each experiment). Data are shown as the mean  +  SD (n = 3 experiments). *p < 0.05 vs the value of "vector 24 h". **(D, E) **Dram1 is not involved in etoposide-induced conventional autophagy. **(D)** shDram1-transfected and control WT MEFs were incubated with 10 μM etoposide for 8 h, and Dram1 expression was assessed by qPCR using mRNA. *p < 0.05. **(E) **The indicated WT MEFs were treated with etoposide (10 μM) for the indicated times in the presence or absence of bafilomycin A1 (10 nM), and the expression of each protein was analyzed by western blotting. GAPDH was included as a loading control.

Dram1 was originally reported as a molecule associated with conventional autophagy 8,9,14, and hence we further analyzed the involvement of Dram1 in conventional autophagy. To this end, we expressed Dram1-Flag into wild-type (WT) MEFs, and analyzed the lipid conjugation of microtubule-associated protein light chain 3 (LC3), which occurs during conventional (but not alternative) autophagy. As shown in **Fig. 5A**, LC3-II (a lipid conjugate) formation was observed in Dram1-expressed MEFs, but not in vector-transfected MEFs in the absence of bafilomycin A1 (BafA), which prevents autophagosome-lysosome fusion. Assessment of autophagic flux, a dynamic process of autophagy that can be measured by differences in the levels of LC3-II with/without BafA, confirmed the induction of conventional autophagy by Dram1 (**Fig. 5A**). Puncta formation of GFP-LC3, another marker of conventional autophagy, was also increased in Dram1-expressing MEFs (**Fig. 5B, C**). Interestingly, we observed GFP-LC3 puncta and GFP-LC3/Dram1 colocalized puncta in Dram1-expressing MEFs (**Fig. 5B**). Because of the lysosomal localization of Dram1, these puncta are thought to be autophagosomes/isolation membranes and autolysosomes, respectively. Consistent results were also observed when HeLa cells were used instead of WT MEFs (Suppl. Fig. 5). These data indicated that Dram1 is sufficient to induced conventional autophagy. We also analyzed whether Dram1 is necessary for etoposide-induced conventional autophagy using shDram1-transfected WT MEFs (**Fig. 5D**). As shown in **Fig. 5E**, LC3-II formation, and autophagy flux were unaffected in etoposide-treated WT MEFs irrespective of the reduction of Dram1. Therefore, Dram1 has the potential to activate conventional autophagy, but its role is marginal in etoposide-induced conventional autophagy, at least in WT MEFs. Dram1 was also reported to regulate genotoxic stress-induced apoptosis 8, but we did not observe any such difference by the silencing of Dram1 (Suppl. Fig. 6). Although the suppression levels of Dram1 from Atg5 KO MEFs and WT MEFs were equivalent (**Fig. 2B, 5D**), Dram1 silencing suppressed alternative autophagy (**Fig. 2**), but not conventional autophagy (**Fig. 5**) and apoptosis (Suppl. Fig. 6), suggesting that Dram1 is more important in alternative autophagy than other cellular functions, at least in MEFs.

**Figure 6:**
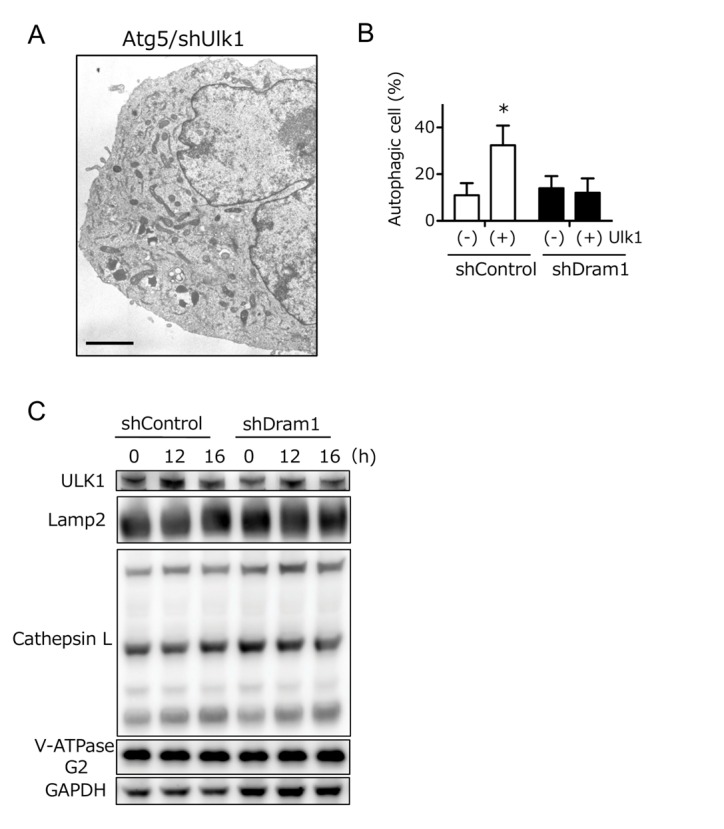
Figure 6: Dram1 functions downstream of Ulk1 in alternative autophagy. **(A)** An electron micrograph of etoposide-treated Atg5 KO MEFs treated with shUlk1 for 18 h. None of the autophagic structures were observed. Bar = 5 μm. **(B)** Suppression of Ulk1-enhanced alternative autophagy by shDram1. shDram1-transfected and control Atg5 KO MEFs were transfected with *ulk1* (1 μg) or a control vector for 24 h. The cells were then treated with etoposide (10 μM) for 8 h and analyzed for immunofluorescence of Lamp2. Percentages of cells with punctate Lamp2 immunofluorescence are shown as the mean + SD (n = 4). *p < 0.05 vs the value of "shControl (−)". **(C)** Normal activation of Ulk1 in ShDram1-transfected Atg5 KO MEFs. shDram1-transfected and control Atg5 KO MEFs were treated with etoposide (10 μM) for the indicated times. The expression level of each protein was analyzed by western blotting. GAPDH was included as a loading control. Ulk1 upregulation was equivalently observed irrespective of the Dram1 silencing.

Ulk1 is another crucial molecule for DNA damage-induced alternative autophagy 6, and hence we studied the association between Ulk1 and Dram1. Ultrastructural analyses demonstrated that etoposide-induced alternative autophagy was blocked before the beginning of isolation membrane formation in Ulk1-silenced Atg5 KO MEFs (**Fig. 6A**). Because Dram1 blocked alternative autophagy by inhibiting the closure of isolation membranes (**Fig. 3A**), Dram1 is thought to function downstream of Ulk1 in the alternative autophagy pathway. This was confirmed by the fact that alternative autophagy was increased by Ulk1 overexpression, but was suppressed by Dram1 silencing (**Fig. 6B**). Furthermore, Ulk1 upregulation, which is essential for alternative autophagy 6, was observed to a similar extent in Dram1-silenced Atg5 KO MEFs (**Fig. 6C** "Ulk1"). Therefore, Dram1 is thought to function at the downstream of Ulk1. Taken together, we here showed the crucial role of Dram1 in the closure of isolation membranes during alternative autophagy.

## DISCUSSION

We previously demonstrated the presence of genotoxic stress-induced alternative autophagy. Although alternative autophagy involves similar morphological features as conventional autophagy, neither Atg5 nor the LC3-conjugation pathway is required. Recently, conventional autophagy was reported to be induced even in the absence of Atg5 in a manner dependent on Syntaxin17. However, genotoxic stress-induced alternative autophagy was not categorized into this type of conventional autophagy, because Syntaxin17 was colocalized with LC3 in starvation-induced conventional autophagy (Suppl. Fig. 7A), but it did not colocalize with alternative autophagic structures in genotoxic stress-treated Atg5 KO MEFs (Suppl. Fig. 7B). Molecularly, we previously reported several crucial molecules, including Ulk1, Beclin1, and Rab9 6, as being involved in this machinery. However, many more molecules are expected to be involved, and our present study demonstrated Dram1 to be one such molecules. Dram1 was first reported as a molecule involved in the induction of conventional autophagy and apoptosis 8,9. However, our study showed that Dram1 also plays a role in alternative autophagy, which might be of greater importance than conventional autophagy and apoptosis, at least in MEFs.

Dram1 has six membrane spanning regions, and largely localizes on lysosomal membranes. In the autophagy pathway, lysosomes function at the final step to generate autolysosomes. If Dram1 functions on lysosomes, it is expected to function at the final step. However, our ultrastructural analyses showed that Dram1 functions at the earlier step of isolation membrane closure. Furthermore, Dram1 did not alter lysosomal protein expression and maturation (**Fig. 6C**). Therefore, alternative autophagy does not appear to be regulated by lysosome-localized Dram1. Instead, Golgi-localized Dram1 appears to be a key player in alternative autophagy. This is because (1) some Dram1 localizes on the Golgi membrane (**Fig. 4**), consistent with a previous report 9, (2) isolation membranes originate from the Golgi membrane, and loss of Dram1 blocked closure of Golgi-derived isolation membranes (**Fig. 2G**), and (3) Dram1-induced autophagosomes were characteristically associated with Golgi membranes (**Fig. 3B**).

Unlike genotoxic stress, Dram1 did not contribute to STS-induced alternative autophagy. This is reasonable because Dram1 is upregulated by genotoxic stress in a p53-dependent manner, and STS does not activate p53. However, because the closure of isolation membranes is necessary for autophagosome generation, additional unidentified molecules are thought to substitute for Dram1. One possible molecule is Dram2, a member of the Dram family that is not induced by p53 15,16. Identification of molecules required for the closure of isolation membranes in STS-induced alternative autophagy will be helpful to elucidate the core machinery of alternative autophagy.

The mechanism as to how overexpressed Dram1 induces alternative autophagy also remains unclear. When we found the induction of large Lamp2 puncta by Dram1 expression, we first considered them as swollen lysosomes, rather than autolysosomes, and assumed that they were generated by Dram1-induced alteration of lysosomal function. However, this idea was denied by ultrastructural analyses, because Dram1-expressing Atg5 KO MEFs contained isolation membranes and autophagosomes enclosing cargos, rather than swollen lysosomes (see **Fig. 3**). Furthermore, Dram1 expression did not alter lysosomal function, as assessed by lysosensor, nor lysosomal protein expression and maturation (Suppl. Fig. 8). Thus, in the case of Dram1 overexpression, enrichment of Dram1 on the Golgi membrane might be the trigger of autophagosome generation, although the precise mechanisms involved are still to be determined.

## MATERIALS AND METHODS

### Reagents

The following antibodies were used for immunoblot and immunofluorescence assays: anti-Lamp2 (Abcam, #ab13524), anti-Flag (Sigma-Aldrich, M2), anti-GS28 (BD Biosciences, #611184), anti-p62 (MBL, PM045), anti-LC3 (NanoTools, #0231-100), anti-Ulk1 (Sigma-Aldrich, A7481), anti-cathepsinL (Santa Cruz, G-11), and anti-V-ATPase G2 (Santa Cruz, A6). Other chemicals were obtained from Nacalai Tesque (Tokyo, Japan).

### Cell culture

Atg5 KO and p53 KO mice have been described previously 17,18. MEFs were generated from Atg5/p53 DKO and Atg5 KO embryos on embryonic day 14.5 by immortalization with the SV40 T antigen 19. Atg5/Ulk1 DKO MEFs were described previously 9. MEFs were grown in modified Dulbecco’s modified Eagle’s medium (DMEM).

For retrovirus transfection, the retroviral plasmids pLMP-shDram1, pLMP-shUlk1, pLMP-Luciferase (shControl), and MSCVhygro-Keima were introduced into MEFs by retroviral infection. Briefly, Plat-E, retroviral packaging cells were plated, and transfected with retrovirus plasmids by the calcium phosphate transfection method 24 hours later. Virus-containing supernatants were collected at 48, 60, and 72 hours post-transfection and MEFs were infected consecutively three times every 12 hours with 4 μg/mL polybrene. Hygromycin (200 μg/mL) or puromycin (2 μg/mL) selection was started 48 hours after the last infection 20. For transient DNA transfection, Lamp1-mCherry and Dram1-Flag plasmids were transfected into MEFs (1 × 10^6 ^cells) using the Neon transfection system (Thermo Fisher Scientific) according to the manufacturer’s instructions.

### Construction of retroviral shRNA vectors

Synthetic oligo shRNAs for the targeted genes were cloned into the retroviral shRNA expression pLMP vector. The following shRNA target sequences were used.

shDram1: AAGAGTTCCTAGTAGTTCAAT; shUlk1: GGGUGGACACAUGCUAAUA; and shLuciferase: ACAAACGCCCTGATCGACAAG.

The siRNA sequences used were as follows: mouse Dram1 5'- AAGAGTTCCTAGTAGTTCAAT -3' and control siRNA (Dharmacon siGENOME Non-Targeting siRNA).

### Electron microscopy

Attached cells were fixed by a conventional method (1.5% paraformaldehyde/3% glutaraldehyde in 0.1 M phosphate buffer at pH 7.4, followed by an aqueous solution of 1% OsO_4_). Fixed samples were embedded in Epon 812, and thin sections (70-80 nm) were then cut and stained with uranyl acetate and lead citrate for observation under a JEOL-1010 electron microscope (JEOL) at 80 kV. Fixed adherent cells were sectioned up to 3 μm from the base. The extent of autophagy was assessed on electron micrographs that contained both the nucleus and cytoplasm of individual cells. The area of every autophagic vacuole and total cytoplasmic area were calculated on the enlarged photographs using a planimeter (Planix). For each cell, the autophagic area (%) was calculated as the total area of autophagic vacuoles relative to the cytoplasmic area, and cells with an autophagic area greater than 6% were defined as autophagic cells (6% was the upper limit in healthy cells). The number of autophagic structures was counted in cells that had a long axis of more than 0.8 μm. Autophagy was quantitated in at least 25 cells for each sample, and was confirmed by two additional independent experiments.

### Correlative light and electron microscopy (CLEM)

To merge the photographs from confocal fluorescence microscopy photo and TEM photo, cells were cultured on coverslips with grids (Matsunami) and were fixed with 1.5% paraformaldehyde/3% glutaraldehyde in 0.1 M phosphate buffer (pH 7.4). Subsequently, the samples were visualized by confocal microscopy; and were fixed using 1% OsO_4 _at 4°C for 15 min and examined by TEM 8.

### Immunofluorescence analysis

Cells were fixed in 4% formaldehyde, permeabilized in 0.1% Triton X-100 or 0.1% saponin PBS, and stained with primary antibodies followed by Alexa Fluor 488 or Alexa Fluor 568 secondary antibodies (Thermo Fisher Scientific). Cells were mounted in mounting medium with propidium iodide or ProLong Gold antifade reagent with DAPI (Thermo Fisher Scientific) and examined by fluorescence microscopy (Zeiss; LSM510 system).

### Quantification of autophagy using Keima and Cyto-ID

Keima-expressing cells were incubated in phenol red-free DMEM. After observing the cells using phase-contrast microscopy, Keima fluorescence was acquired using multiple wavelengths (405 and 555 nm). Ratio (555/405) images of Keima were created and merged with the phase-contrast images. Cells were also stained with Cyto-ID autophagy dye (Enzo) for 30 min at 37°C, followed by fixation in 4% formaldehyde. Cells were mounted in ProLong Gold antifade reagent with DAPI (Thermo Fisher Scientific) and examined using a confocal microscope (Zeiss; LSM510 system). The extent of Cyto-ID fluorescence was also analyzed using a flow cytometer (FACS Canto).

### Quantitative RT-PCR and microarray analysis 

For microarray analysis, total RNA was purified using TRIzol reagent (Invitrogen) and utilized for preparing cRNA by Message AmpII (Ambion). cRNA was hybridized to Mouse Whole Genome 4 × 44 K microarrays (Agilent Technologies) according to the manufacturer’s instructions. Two biological replicates were performed for each set of experimental conditions. Data were analyzed using Feature Extraction Software (ver. 9.5.3.1) (Agilent). For quantitative RT-PCR analysis, total RNA was purified on an RNeasy column (Qiagen) and utilized for mRNA purification by Turbo Capture8 (Qiagen). Synthesis of cDNA and PCR amplification were performed using the iScript One-step RT-PCR kit with SYBR Green (Bio-Rad Laboratories). Quantitative analysis was performed with the CFX96 real-time PCR system (Bio-Rad). All samples were normalized using ß-actin levels.

### Statistical analysis

Results are expressed as the mean + standard deviation (SD). Statistical analysis was performed using Prism (GraphPad) software. Comparisons of multiple datasets were performed using one-way ANOVA followed by Tukey’s *post hoc* test. A *p*-value of less than 0.05 was considered to indicate a statistically significant difference between two groups. Statistical analyses of nonrandom associations between two categorical variables were analyzed using the Fisher’s exact test.

## SUPPLEMENTAL MATERIAL

Click here for supplemental data file.

All supplemental data for this article are also available online at http://www.cell-stress.com/researcharticles/dram1-regulates-dna-damage-induced-alternative-autophagy/.

## Abbreviations

Atg: autophagy related
CLEM: correlative light electron microscopy
DKO: double knock out
Dram1: dagame-regulated autophagy modulator 1
EM: electron microscopy
KO: knock out
LC3: light chain 3
MEF: mouse embryonic fibroblast
shRNA: short hairpin RNA
STS: staurosporine
WT: wild type
