# Supplementary material for: Dram1 regulates DNA damage-induced alternative autophagy
Source: Cell Stress. 2018 Mar 7;2(3):55–65. doi: 10.15698/cst2018.03.127 (PMC6558930; doi:10.15698/cst2018.03.127)
Supplement: Supplementary file 1 [file ces-02-055-s01.pdf]

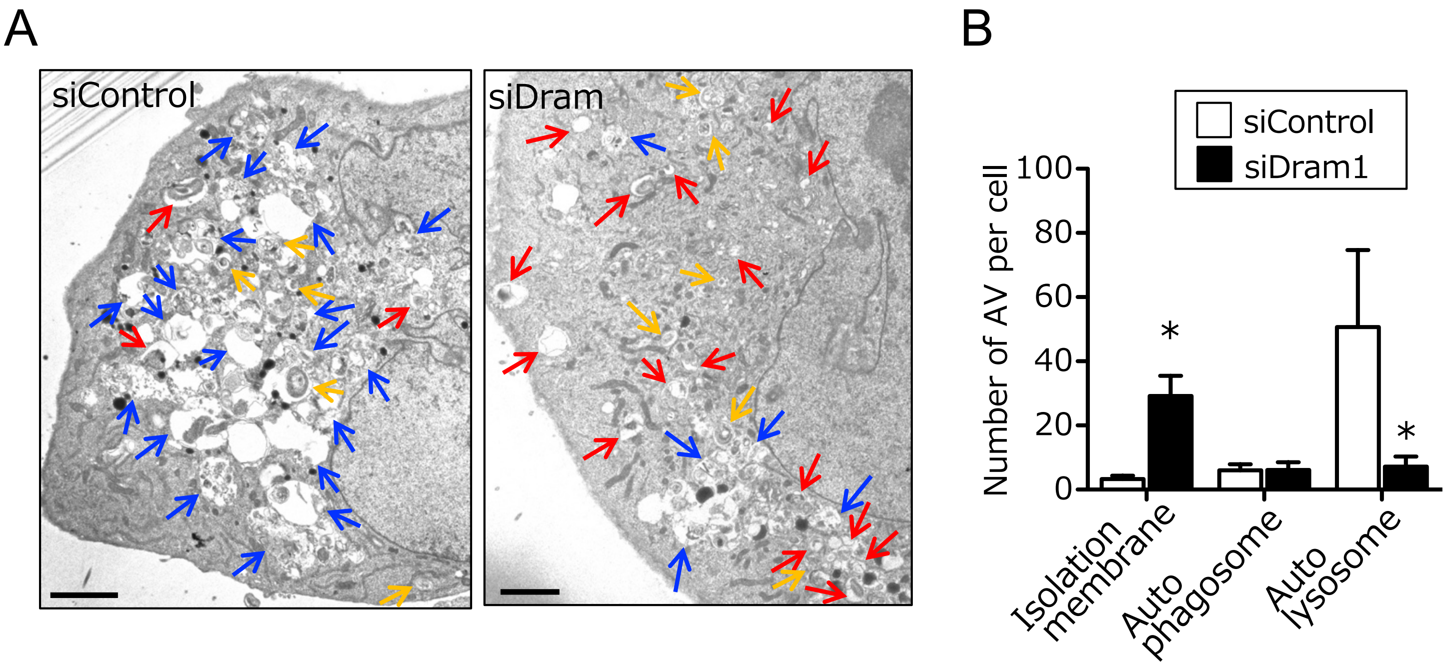

Suppl. Figure 1. Inhibition of alternative autophagy in etoposide-treated Atg5 KO MEFs by an siRNA for Dram1

Electron micrographs of siDram1-transfected and siControl-transfected Atg5 KO MEFs treated with etoposide (10 µM) for 18 h. In **(A)**, blue, yellow, and red arrows indicate autolysosomes, autophagosomes, and isolation membranes, respectively. Bar = 2 µm. **(B)** The number of each type of autophagic structure appearing in Atg5 KO MEFs treated with etoposide. Dram1 siRNA-transfected and control Atg5 KO MEFs were incubated with 10 µM etoposide for 18 h. The number of autophagic vacuoles per cell was counted on the EM photographs. White and black columns represent the number of autophagic structures in control and siDram1-transfected Atg5 KO MEFs, respectively. Data are the mean + SD obtained from 10 cells. *$p < 0.05$ vs the value of "siControl".

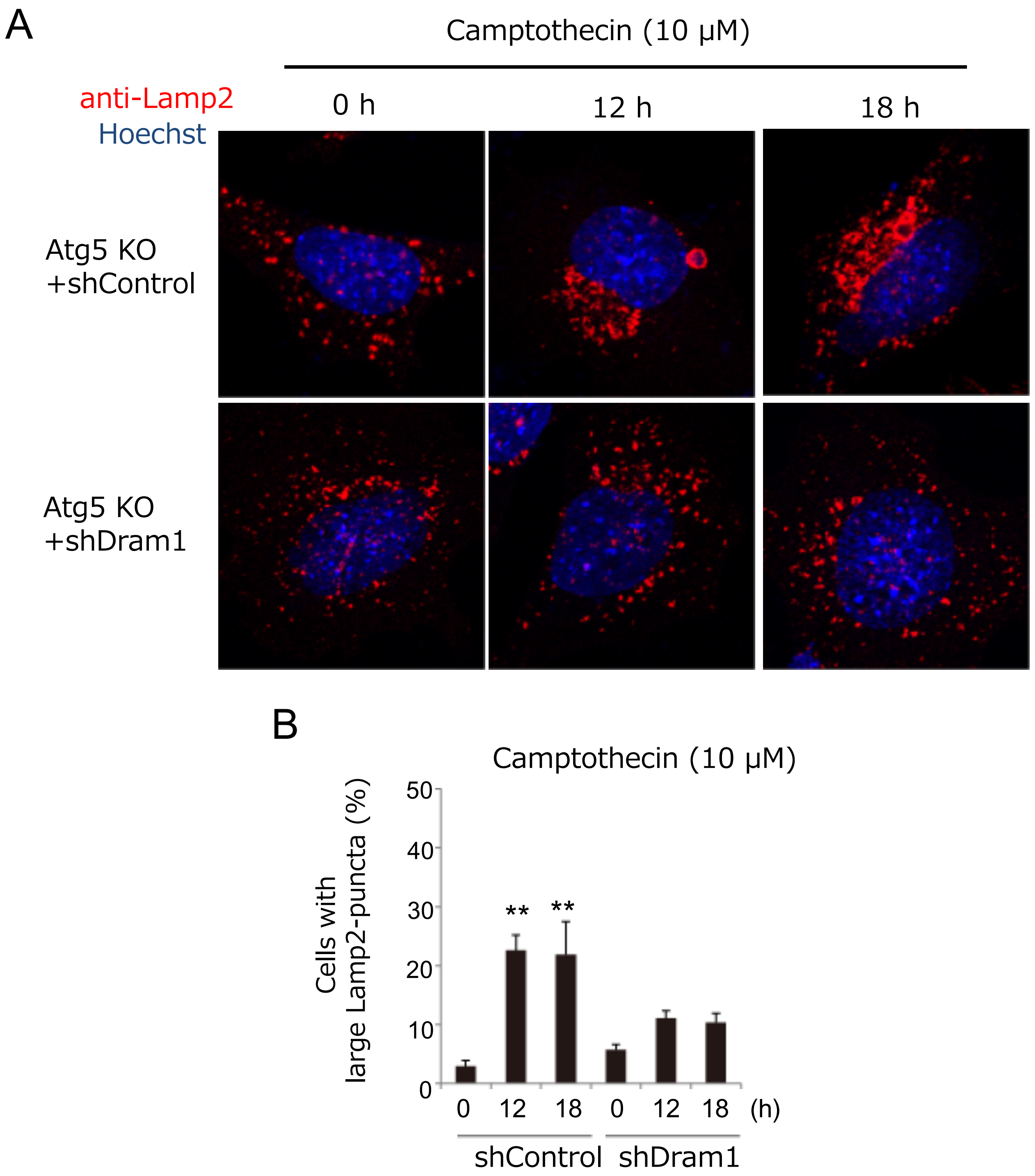

Suppl. Figure 2. Involvement of Dram1 in camptothecin-induced alternative autophagy

The indicated Atg5 KO MEFs were treated with camptothecin (10 μM) for the indicated times. Alternative autophagy was then analyzed using Lamp2 immunofluorescence. **(A)** Representative images are shown. **(B)** Percentages of large Lamp2 puncta-containing cells are shown as the mean + SD (n = 4). $^{**}p < 0.01$ vs the value of "shControl 0 h".

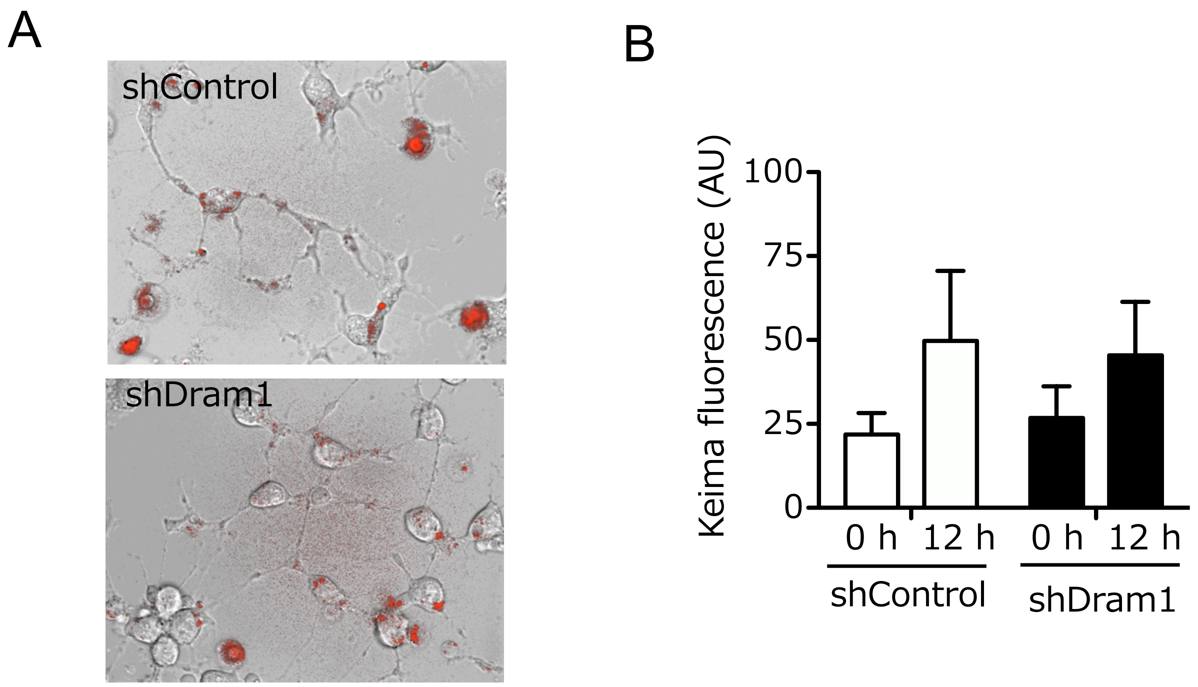

Suppl. Figure 3. Dram1 does not play a role in staurosporine-induced alternative autophagy

The indicated Atg5 KO MEFs were treated with staurosporine (1 μM) for 12 h. Alternative autophagy was then examined using Keima. **(A)** Keima signals (red) are merged with images obtained from phase-contrast microscopy at 12 h. **(B)** The extent of the Keima signals is shown as the mean + SD (n = 4).

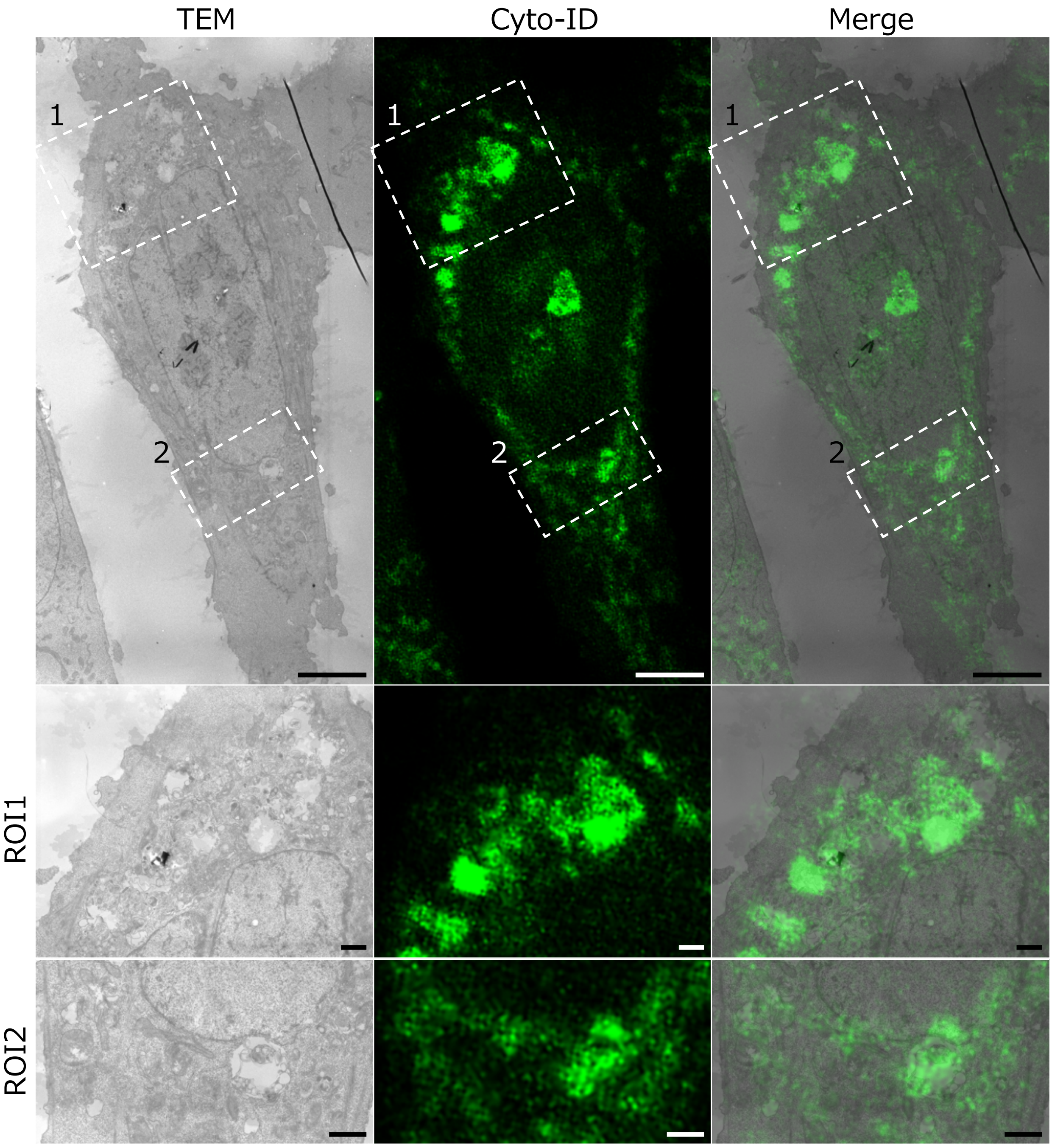

Suppl. Figure 4. Identification of autophagic vacuoles as Cyto-ID-positive structures by CLEM

Atg5 KO MEFs were treated with 10 µM of CBM, an inducer of alternative autophagy, and were stained with Cyto-ID using coverslips with grids and fixed with glutaraldehyde and paraformaldehyde. After Cyto-ID signals were assessed, the cells were further fixed with OsO_4 and examined by EM (TEM). Two regions of interest (ROI) are indicated by dashed squares and their magnified images are shown in the bottom panels. Cyto-ID-positive structures (green signals) were identical to accumulated autophagic vacuoles.

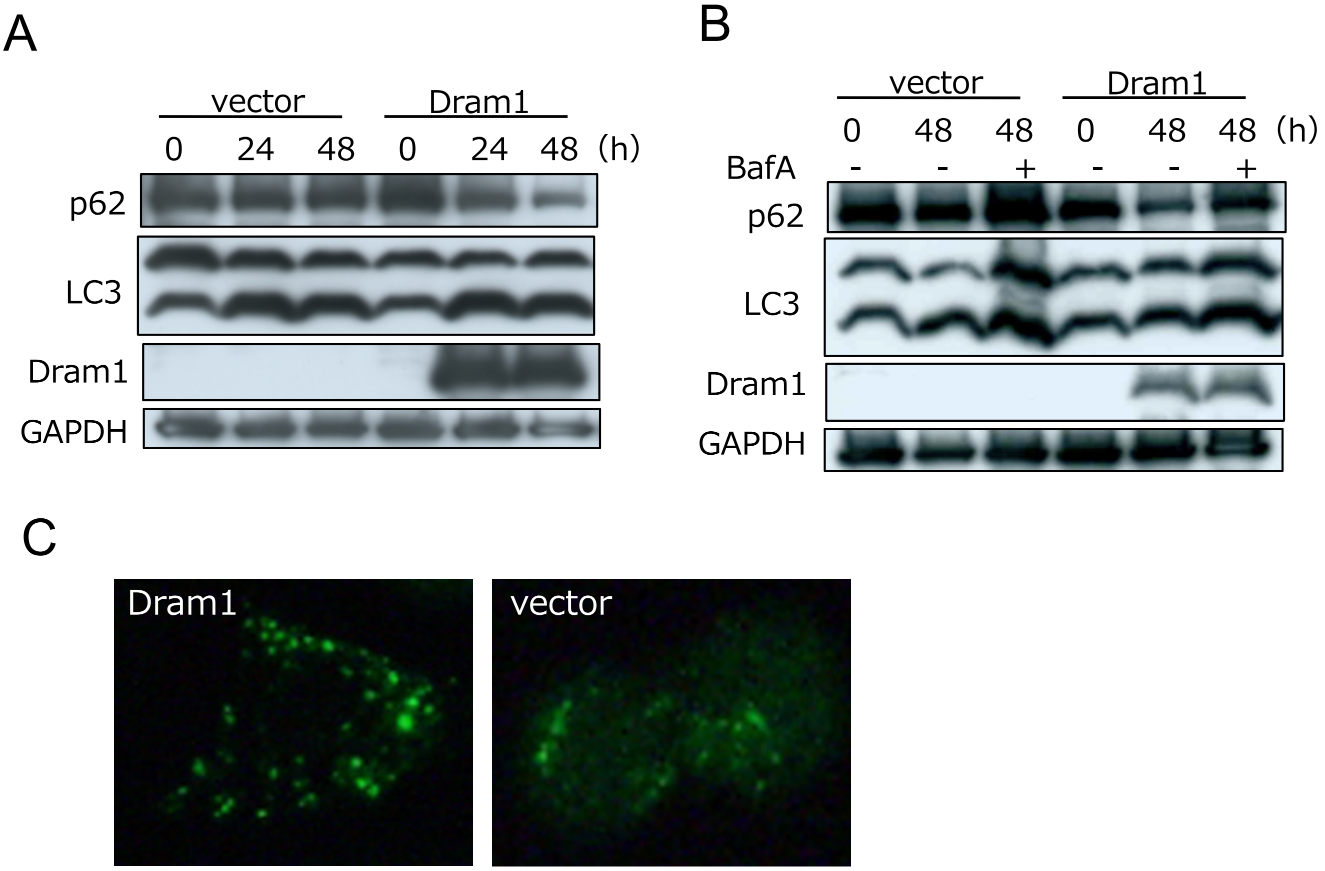

Suppl. Figure 5. Dram1 is sufficient to induce conventional autophagy in HeLa cells

(A, B) HeLa cells were transfected with *dram1-flag* or a control vector for the indicated times in the presence or absence of bafilomycin A1 (BafA; 10 nM), and the expression of each protein was analyzed by western blotting. GAPDH was included as a loading control. **(C)** GFP-LC3-expressing HeLa cells were transfected with *dram1-flag* or a control vector, and then their GFP-LC3 signals (green) were observed. Representative images are shown.

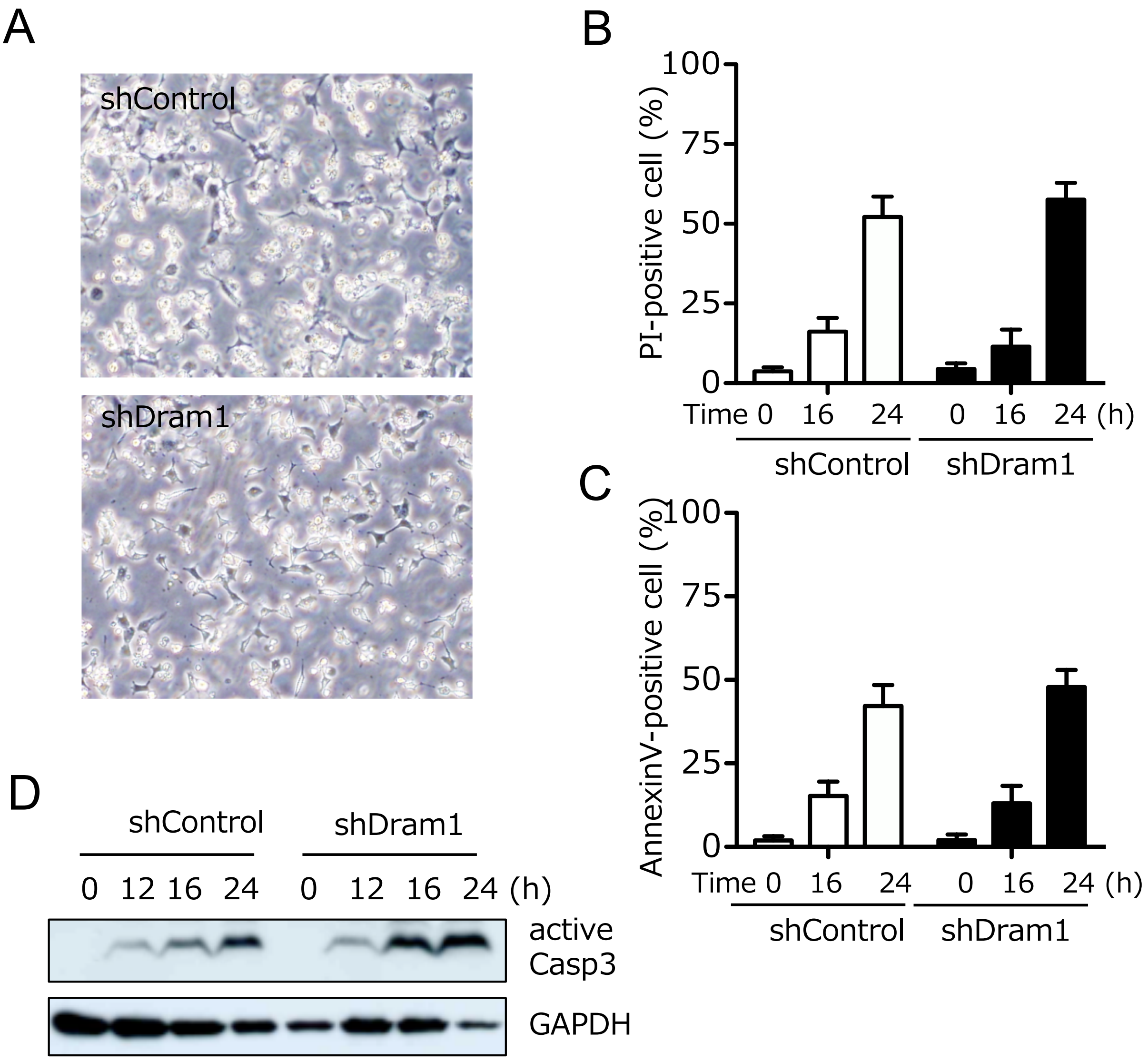

Suppl. Figure 6. Dram1 is not involved in etoposide-induced apoptosis of Atg5 KO MEFs

shDram1-transfected and control Atg5 KO MEFs were incubated with 20 μM etoposide for the indicated times, and cell death was analyzed by phase-contrast imaging (at 24 h) **(A)**, propidium iodide staining **(B)**, AannexinV staining **(C)**, and western blotting for active caspase-3 (Casp3) **(D)**. Apoptosis was induced irrespective of the presence of Dram1.

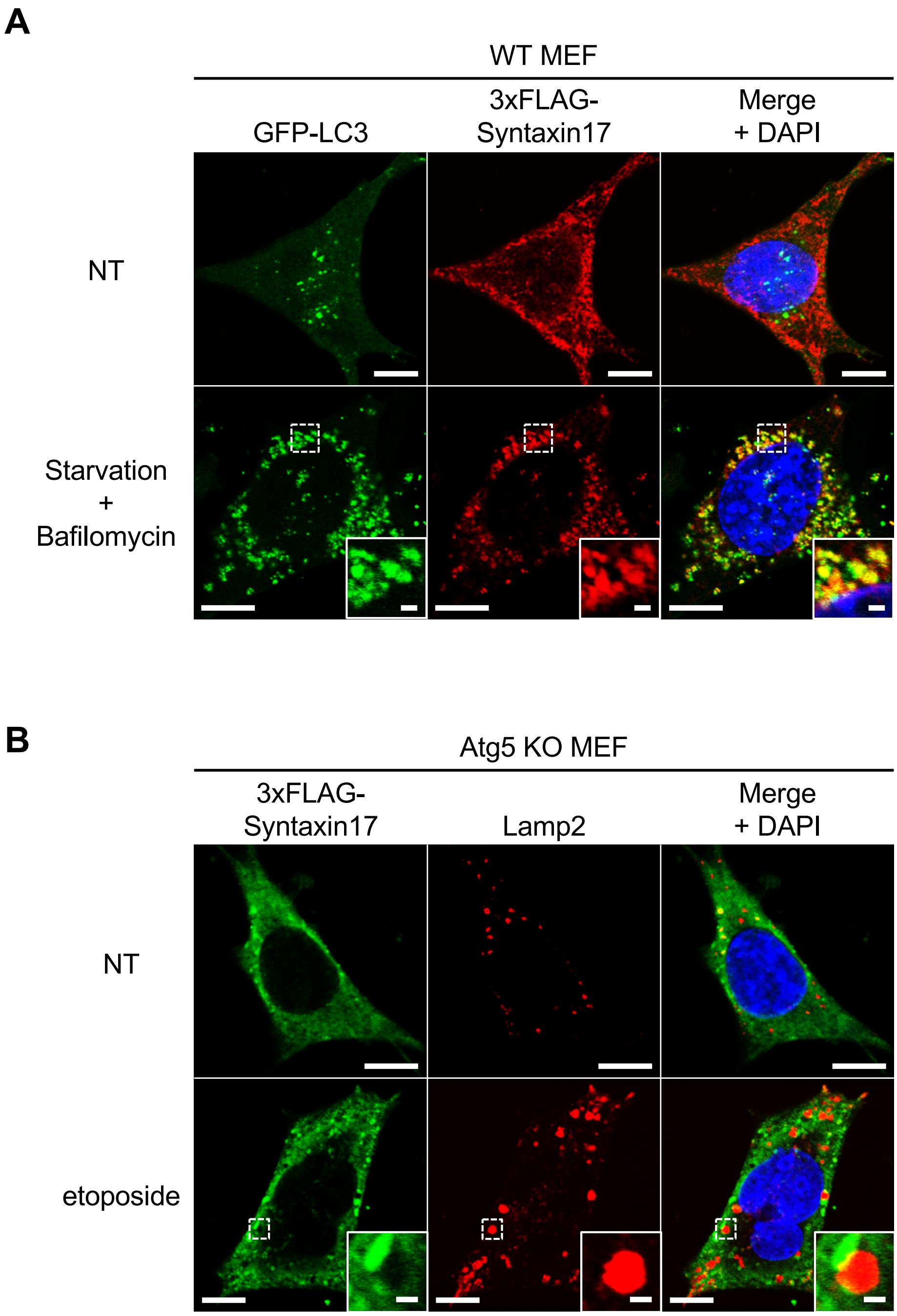

Suppl. Figure 7. Syntaxin17 is not involved in etoposide-induced alternative autophagy

(A) WT MEFs expressing GFP-LC3 and Flag-Syntaxin17 were left untreated (NT) or were starved (6 h), and conventional autophagy was analyzed using GFP-LC3. Then, cells were immunostained with an anti-Flag antibody (red). Representative images are shown (bars = 10 μm). ROIs are indicated by the dashed squares and magnified images are shown in the inset (bars = 1 μm). GFP-LC3 puncta were well merged with Syntaxin17.
(B) Atg5 KO MEFs expressing Flag-Syntaxin17 were treated with etoposide (10 μM for 18 h), and cells were immunostained with anti-Flag (green) and anti-Lamp2 (red) antibodies. Representative images are shown (bars = 10 μm). ROIs are indicated by the dashed squares and magnified images are shown in the inset (bars = 1 μm). Large Lamp2-puncta were not merged with Syntaxin17.

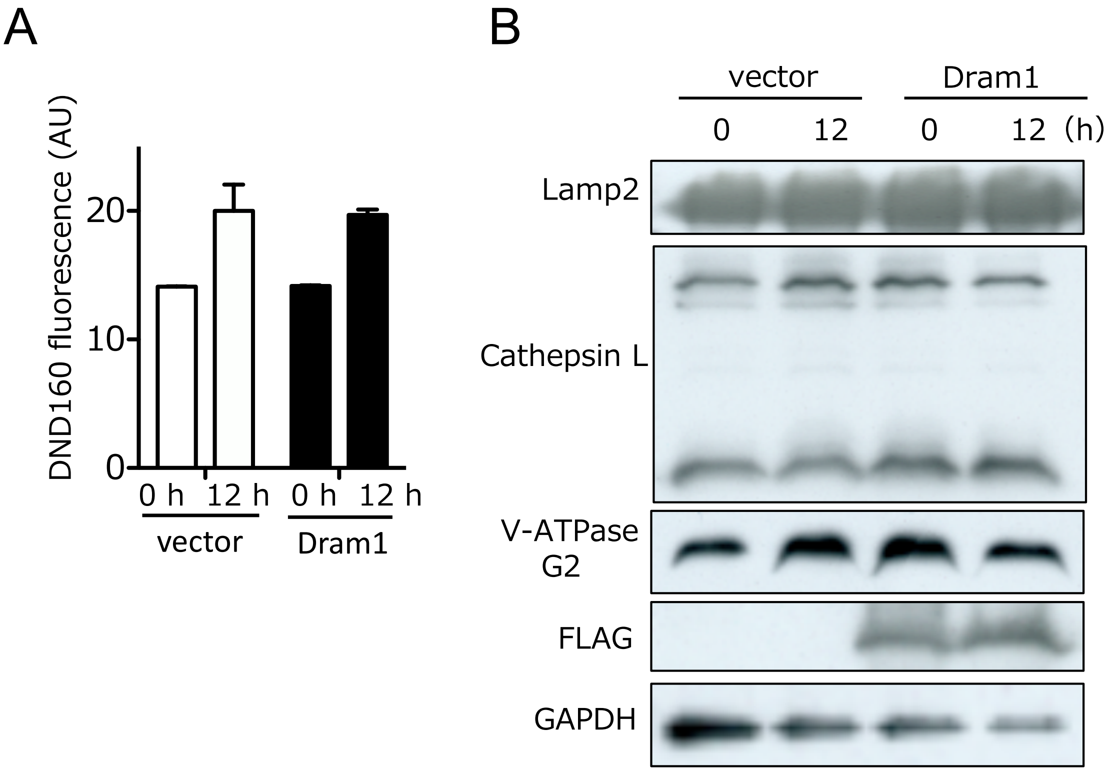

Suppl. Figure 8. Dram1 does not affect lysosomal function

Atg5 KO MEFs were transfected with *dram1-flag* or a control vector for the indicated times. **(A)** Then, lysosomal function was analyzed using lysosensor DND160. **(B)** The expression of lysosomal proteins was analyzed by western blotting. GAPDH was included as a loading control. Lysosomal function was not affected by the expression of Dram1.
